# Reprogramming of regulatory T cells in inflammatory tumor microenvironment: can it become immunotherapy turning point?

**DOI:** 10.3389/fimmu.2024.1345838

**Published:** 2024-02-21

**Authors:** Jinming Liu, Biao Zhang, Guolin Zhang, Dong Shang

**Affiliations:** ^1^ Department of General Surgery, Clinical Laboratory of Integrative Medicine, The First Affiliated Hospital of Dalian Medical University, Dalian, China; ^2^ Department of Cardiology, The Second Hospital of Dalian Medical University, Dalian, China; ^3^ Institute (College) of Integrative Medicine, Dalian Medical University, Dalian, China

**Keywords:** regulatory T cells, reprogramming, tumor immunity, Foxp3, metabolize, posttranslational modification, cancer therapy

## Abstract

Overcoming the immunosuppressive tumor microenvironment and identifying widely used immunosuppressants with minimal side effects are two major challenges currently hampering cancer immunotherapy. Regulatory T cells (Tregs) are present in almost all cancer tissues and play an important role in preserving autoimmune tolerance and tissue homeostasis. The tumor inflammatory microenvironment causes the reprogramming of Tregs, resulting in the conversion of Tregs to immunosuppressive phenotypes. This process ultimately facilitates tumor immune escape or tumor progression. However, current systemic Treg depletion therapies may lead to severe autoimmune toxicity. Therefore, it is crucial to understand the mechanism of Treg reprogramming and develop immunotherapies that selectively target Tregs within tumors. This article provides a comprehensive review of the potential mechanisms involved in Treg cell reprogramming and explores the application of Treg cell immunotherapy. The interference with reprogramming pathways has shown promise in reducing the number of tumor-associated Tregs or impairing their function during immunotherapy, thereby improving anti-tumor immune responses. Furthermore, a deeper understanding of the mechanisms that drive Treg cell reprogramming could reveal new molecular targets for future treatments.

## 1 Introduction

Immune checkpoint blockade therapy has shown significant advancements in cancer treatment. However, it is important to recognize that this treatment can only cure a limited proportion of patients, while the majority may not benefit from it and could potentially experience autoimmune diseases and unforeseen toxicities (1, 2). To address these challenges, we can focus on two key aspects: overcoming the immunosuppressive tumor microenvironment and improving local-specific antitumor immunity (3–5). One potential target to achieve these goals is Tregs. Additionally, cancer is strongly influenced by the presence of immune cells and the levels of inflammatory cytokines. In tissues experiencing inflammation, they are also important in avoiding severe tissue injury (6, 7). However, immune cell malfunctions can cause inflammation to advance into cancer.

Regulatory T cells (Tregs) are recognized as a specific subset of CD4+ T cells. Foxp3 is a unique transcription factor (8). According to their origin, we can divide them into thymic Tregs (tTregs)/natural Tregs (nTregs) and peripheral Tregs (pTregs)/inducible Tregs (iTregs) (9). Abul K Abbas et al. proposed a unified classification of Tregs into tTregs and pTregs (10). The tTregs are produced by the thymus and are tissue-intrinsic Tregs. After being stimulated by self-antigens, they are transported to the periphery and exhibit inhibitory activity against self-antigens (11, 12). pTregs are differentiated from naïve T cells or conventional T cells in peripheral tissues, or induced by TGF-β *in vitro*. pTregs play a role in preventing autoimmunity caused by foreign antigens (13). According to the function and phenotype of Tregs, they are divided into naïve Tregs (or resting Tregs), effector Tregs (eTregs), and non-Tregs. Naïve Tregs manifested as CD4^+^FOXP3^low^ CD25^hi^Tregs, eTregs manifested as CD4^+^FOXP3^hi^ CD25^hi^Tregs, and non-Tregs manifested as CD4^+^FOXP3^low^ Tregs (14). Under steady-state conditions, naïve Tregs have weak immunosuppressive functions and eTregs have strong immunosuppressive functions. Non-Tregs have no suppressive function and secrete pro-inflammatory factors (14). In the inflammatory environment, nonTregs are also known as “exTreg” or “exFoxp3” cells (15).

In the tumor microenvironment (TME), naïve Tregs and exTregs will be converted into tumor-infiltrating Tregs (TI-Tregs) under various stimuli. TI-Tregs have the same phenotype as eTregs (14). This process is called Tregs reprogramming. Infiltration of reprogrammed Tregs into tumors is associated with a negative prognosis (16). Thus, targeting the reprogramming process of Tregs and paying attention to their altered state in both inflammation and cancer could potentially provide new perspectives for cancer immunotherapy. Therefore, this article aims to deeply explore the mechanism of reprogramming exTregs into immunosuppressive Tregs.

Recent research has shown that specific signaling molecules found within the tumor microenvironment, along with factors generated by immune cells and tumor cells, have the potential to initiate the reprogramming of exTregs (17, 18). In the hypoxic, low-glucose, and high-lactic acid conditions of the tumor microenvironment, exTregs undergo metabolic adaptability through reprogramming, gradually transitioning towards an immunosuppressive phenotype (19–21). Furthermore, Foxp3, the most crucial transcription factor regulating Tregs, undergoes post-translational modification involving methylation, acetylation, glycosylation, phosphorylation, and ubiquitination. The function and reprogramming of Tregs rely heavily on these regulatory mechanisms (9, 22, 23).

This work aims to present a summary of the mechanism of reprogramming exTregs into eTregs, from the following three aspects: (1) signaling molecules and intracellular signaling pathways in the tumor microenvironment; (2) metabolic and energy pathways including glycolysis, amino acid metabolism, fatty acid oxidation (FAO) and mevalonate pathways, mitochondrial oxidative phosphorylation and complexes (OXPHOS), and metabolite pathways in TME; (3) the modification of Foxp3 expression at the transcriptional level, such as methylation, acetylation, glycosylation, phosphorylation, and ubiquitination of Foxp3, affects the immunosuppressive function of Tregs.

## 2 Immunosuppressive immune cells within TME

Targeting specific immune cells, especially Tregs, and inhibiting their immunosuppressive functions is a crucial approach in current immunotherapy. Additionally, other immune cells such as regulatory B cells (Bregs), regulatory dendritic cells (DCregs), myeloid-derived suppressor cells (MDSCs), and tumor-associated macrophages (TAMs) also possess immunosuppressive capabilities (24). Bregs, which constitute only 10% of circulating B cells in healthy individuals, exert their suppressive effects on autoreactive B cells through the secretion of soluble molecules and the expression of inhibitory molecules. However, due to the lack of a specific transcription factor defining Bregs, there is currently limited research in this area (25). DCregs, which are subpopulations of DCs in central and peripheral lymph, exhibit lower levels of MHC and costimulatory molecules on their surface. DCregs exert immunosuppressive functions mainly by inducing the inactivation of autoreactive T cells and Tregs differentiation, and increasing the levels of inhibitory molecules and cytokines such as IL-10 (24, 26). Similarly, MDSCs, known for their immunosuppressive function, inhibit T cell responses, induce Tregs, and differentiate into TAMs (27, 28). TAMs have low expression of inhibitory molecules such as MHCII and PD-1, thereby exerting immunosuppressive effects and promoting tumor growth (29, 30).

## 3 The reprogramming mechanism of exTregs to eTregs

Under normal circumstances, Tregs exhibit stability. However, in specific conditions like an inflammatory tumor microenvironment, certain Tregs undergo reprogramming (31). In most cancers, there is infiltration of eTregs, while in some cancers, both eTregs and exTregs are present (32). This review focuses on elucidating the mechanism of exTregs reprogramming into eTregs from three distinct perspectives.

### 3.1 Signaling molecules and pathways

**Figure 1:**
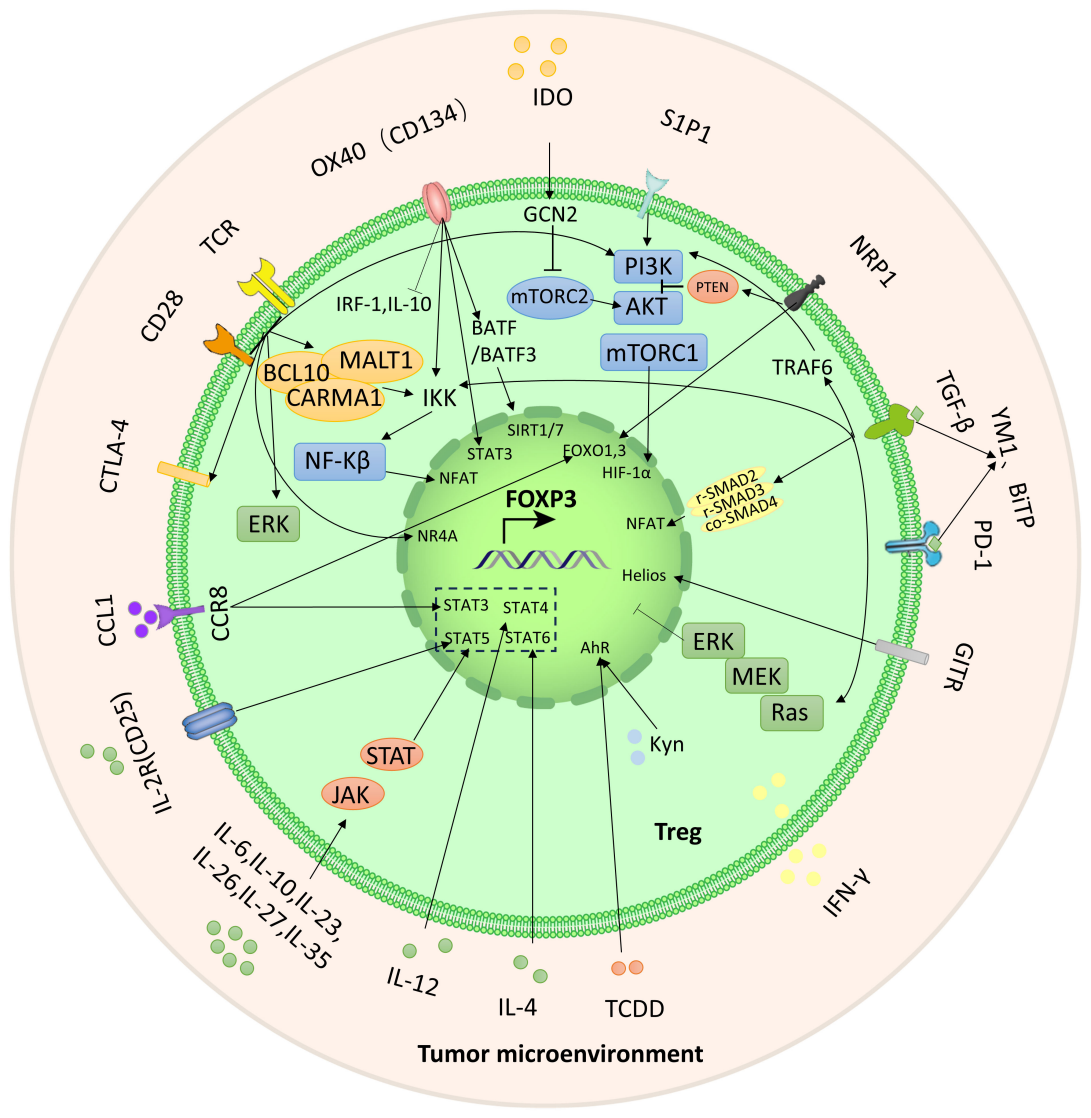
The crucial signals and pathways for the reprogramming of Tregs. Reprogramming of Tregs from inflammatory to immunosuppressive activity is achieved through these signaling molecules and pathways (as highlighted by the lines and arrows in the figure).

Recent research has demonstrated that certain signaling molecules within TME can cause exTregs to undergo reprogramming (**Figure 1**). This reprogramming process can lead to a transition of exTregs from their typical immunoregulatory state to a state that promotes tumor growth.

#### 3.1.1 TCR/CD28

The leukocyte surface differentiation antigen 28 (CD28) costimulatory receptor forms microclusters with the T cell receptor (TCR) and plays a dual role in the reprogramming of Tregs (33). The TCR-CD28 microclusters trigger various cytoplasmic signaling pathways, such as the induction of the CARMA1-BCL10-MALT1 (CBM) complex. The CBM complex further stimulates IKK-mediated NF-κB activation, which subsequently activates the transcription factor NFAT and promotes immunosuppression (34–37). In addition, TCR/CD28 costimulation can induce cytotoxic T-lymphocyte antigen-4 (CTLA-4). The high expression of CTLA-4 on Tregs supports its immunosuppressive activity, so anti-CTLA-4 antibody has been developed as an immune checkpoint inhibitor (38, 39).

Notably, TCR/CD28 can also subvert immunosuppression, primarily through the phosphatidylinositol 3-kinase (PI3K)-AKT-mammalian target of the rapamycin (mTOR) pathway. It is also crucial for metabolism. In addition to TCR/CD28, sphingosine-1-phosphate receptor 1 (S1PR1) and interleukin-2 receptor (IL-2R) can also activate the PI3K-AKT-mTOR1 signaling pathway (40, 41). AKT can inhibit the activity of transcription factors FOXO1 and FOXO3 and inhibit the expression of Foxp3 (42). mTORC1 can bind with the transcription factor known as hypoxia-inducible factor (HIF-1α). Additionally, HIF-1α exerts inhibitory effects on the expression of Foxp3 (43, 44). Studies by Sharma et al. have shown that neuropilin-1 (Nrp1) in Tregs can inhibit this pathway through phosphatase and tensin homolog (PTEN), and lead to increased nuclear translocation of FOXO1 and FOXO3a resulting in increased Foxp3 expression and enhanced immunosuppression (45, 46). In addition, TCR/CD28 costimulation can also affect the expression of Foxp3 through the nuclear receptor Nr4a, extracellular signal-regulated kinase (ERK), Ca2+, and protein kinases PKA and PKC (47, 48).

#### 3.1.2 Transforming growth factor beta

TGF-β exerts its influence on Treg function through both Smad-dependent and Smad-independent pathways, which may have different effects in different contexts. Smad protein serves as a downstream effector transcription factor in the TGF-β signaling pathway. R-Smad is activated when activated TGF-β interacts with its receptor. R-smad2 and r-smad3 form a complex with co-smad4 and bind to the CNS1 region of Foxp3 through the transcription factor NFAT, thereby promoting Foxp3 expression (49, 50).

Apart from the Smad-dependent signaling pathway, the activated TGF-β receptor (TGFβR) can recruit TAK1 (TGF-β-activated kinase 1) to further activate the NF-κB pathway and enhance Foxp3 expression (51). Moreover, TGF-β stimulates the PI3K-AKT-mTOR pathway through the interaction of activated TGFβRI and TGFβRII with p85 (the regulatory unit of PI3K), then inhibits Foxp3 expression (52). Nrp1-activated PTEN can block the PI3K-AKT-mTOR pathway. Moreover, TGFβR assembles the ShcA-Grb2-SOS complex by phosphorylating ShcA and activates the Ras-MEK-ERK pathway, and the MEK-ERK-dependent pathway can inhibit Foxp3 expression (53). Through the TRAF6-mediated pathway, TGF-β induces ubiquitination of p85, thereby initiating the activation of AKT-mTOR and inhibiting Foxp3 expression (50).

Disruption of TGF-β signaling underlies inflammatory diseases (54). In the early stages of cancer, TGF-β induces apoptosis in precancerous cells and inhibits tumor cell proliferation. As tumors progress, tumor-derived TGF-β can trigger tumorigenic and pro-metastatic responses in cancer cells and stroma, including the formation of an immunosuppressive tumor microenvironment (55, 56). At this time, TGF-β can affect a variety of immune cells, including inhibiting the proliferation and function of T cells, inducing the differentiation of naïve T cells into regulatory T cells, etc (54). At present, great progress has been made in immunotherapy targeting TGF-β-mediated immunosuppression, including TGF-β mRNA-directed agents, ligand traps, antibodies, fusion proteins and small-molecule kinase inhibitors of TGFβRs (57). Mouse model studies have shown a strong synergistic effect between TGF-β pathway inhibitors and ICIs (58). In particular, the anti-PD-L1/TGF-βR bispecific antibodies YM1 and BiTP can effectively inhibit the effect of TGF-β-Smad, have better anticancer effects than antibodies that antagonize TGF-β alone, and can also restore the response to PD- L1 drug resistance (59–62).

#### 3.1.3 IL family and transcription factor STAT family

As a family of cytokines, the interleukin (IL) family is important in TME. According to the homology of cytokines, the IL family can be divided into IL-1, IL-2, IL-6, IL-10, IL-12, IL-17 families, etc. IL-33 and IL-36, which are part of the IL-1 family, play a role in the activation of Tregs (63, 64). IL-36γ signals through the IL-36R, MyD88, and NFκBp50 in CD4^+^ T cells, effectively inhibiting the development of Foxp3-expressing Tregs (64). The STAT family, a transcriptional activator, could control the expression of Foxp3. A significant correlation between IL family and STAT family has been observed (65–67). IL-2 is crucial for Treg function. The combination of IL-2 and IL-2R phosphorylates and activates the transcription factor STAT5, which in turn increases Foxp3 expression (68–70). CD25, a component of IL-2R, can be targeted by daclizumab to effectively reduce the expression of Foxp3 and increase the secretion of IFN-γ (71, 72). This phenomenon could potentially occur via the IFN-γ-mediated polarization of Tregs towards Th1-like effector T cells (Teffs) (73). Additionally, IL-2 is also required for CD8+ T cells to remain activated and continue to exhibit cytotoxic effects. Tregs, which have high IL-2R expression, impede the activation of Teffs and induce apoptosis of these cells, thereby mediating immune suppression (74, 75). Furthermore, IL-4 activates STAT6 and inhibits the transcription of Foxp3, while IL-12 has been found to regulate STAT4 and inhibit the expression of Foxp3 (76–79).

It is worth noting that IL-6, IL-10, IL-23, IL-26, IL-27, and IL-35 can regulate STAT3 and participate in the JAK-STAT pathway, which in turn affects the expression of Foxp3 (80–85). This suggests that STAT3 can be activated by various cytokines, leading to an impact on Tregs. The effects of certain factors on Tregs can be the opposite, such as the pro-inflammatory properties of IL-6 and the anti-inflammatory properties of IL-10. This suggests that STAT3 may play different roles in Tregs (80). Among these factors, IL-26 is a constituent of the IL-10 family, while IL-23, IL-27, and IL-35 are part of the IL-12 family. The influence of IL-27 on the promotion or suppression of Tregs is influenced by multiple factors and requires further investigation (84).

#### 3.1.4 Glucocorticoid-induced tumor necrosis factor receptor

Glucocorticoid-induced tumor necrosis factor receptor (GITR), a TNFR, acts as a costimulatory molecule and has high expression levels on Tregs. Antibodies targeting GITR have shown promising antitumor effects. These antibodies not only reduce the number of Tregs but also disrupt the reprogramming of Tregs and their immunosuppressive function (86–88). In the inflammatory tumor microenvironment, the transcription factor Helios plays an important role in maintaining the stability of Tregs (89, 90). Treatment with anti-GITR drugs decreases Helios expression, consequently downregulating Foxp3 expression, as well as reducing IL-10 levels and increasing IFN-γ levels (91–93).

#### 3.1.5 OX40

In addition to TCR signaling, the TNF receptor OX40 can also activate IκB kinase β (IKKβ), causing c-Rel and RelA to be translocated to the nucleus. This activation participates in the NF-kB pathway and regulates Foxp3 expression (51, 94). Furthermore, OX40 can activate the Akt and Stat5 pathways, causing temporary proliferation of Tregs and reducing Foxp3 expression levels. However, this will lead to a relative deficiency of IL-2. The use of IL-2 agonists can rescue this situation, enabling Tregs to exert their immunosuppressive function (95). OX40 can also up regulate the expression of BATF and BATF3 in CD4^+^T cells, and inhibit the expression of Foxp3 by histone deacetylases SIRT1/7 (96). In addition, under the action of OX40 agonist, the expression of transcription factor interferon regulatory factor 1(IRF1) and the production of IL-10 of Tregs were inhibited, inhibiting the immunosuppressive function of Tregs (97, 98).

#### 3.1.6 Aryl hydrocarbon receptor

2,3,7,8-tetrachlorodibenzo-p-dioxin (TCDD), an environmental pollutant, exhibits a strong affinity for the AhR (99). AhR serves as a crucial transcription factor that regulates genes associated with inflammation and subsequently influences Tregs. According to recent scientific investigations, it has been uncovered that TCDD possesses the capability to stimulate AhR. Consequently, AhR forms a connection with the DRE sequence situated within the Foxp3 promoter, effectively augmenting the expression of Foxp3 (99, 100). L-kynurenine (Kyn), a tryptophan metabolite of indoleamine 2,3-dioxygenase (IDO), interacts with AhR to enhance the expression of Foxp3 (101).

#### 3.1.7 Cdc42 GTP enzyme

Cdc42 is a Rho family GTPase. Recent studies have shown that inhibiting Cdc42 GTPase or its direct downstream effector WASP can disrupt the reprogramming of Tregs and stimulate anti-tumor immunity. In terms of mechanism, inhibition of Cdc42 and its direct downstream effector WASP works by promoting the non-canonical signaling cascade GATA-binding protein 3 (GATA3)-carbonic anhydrase I (CAI). CAI-mediated pH changes induce anti-tumor T cell immunity (102).

#### 3.1.8 IDO signaling

Under inflammatory conditions, dendritic cells (DCs) signal IDO, which reduces tryptophan concentrations near Tregs. The decline in tryptophan levels triggers the activation of GCN2 kinase, which hinders the functioning of the mTORC2 complex and impedes its phosphorylation of Akt at Ser473 (45, 103). The signaling of Akt in Tregs leads to the impairment of their immunosuppressive function (104–106). IDO signaling inhibits Akt, upregulates transcription factor FOXO3a, and promotes Treg reprogramming into an immunosuppressive phenotype.

#### 3.1.9 Chemokine receptor 8

The CC chemokine CCL1 receptor CCR8 is selectively expressed on Tregs in TME and is associated with poor tumor prognosis (107). The CCR8-CCL1 pathway can increase STAT3 expression and FOXO1 entry into the nucleus, thereby increasing Foxp3 expression. In addition, CCR8-CCL1 can also increase the expression of CD39 and IL-10, jointly promoting Treg reprogramming and enhancing immunosuppressive ability (108, 109).

### 3.2 Metabolic and energy pathways

**Figure 2:**
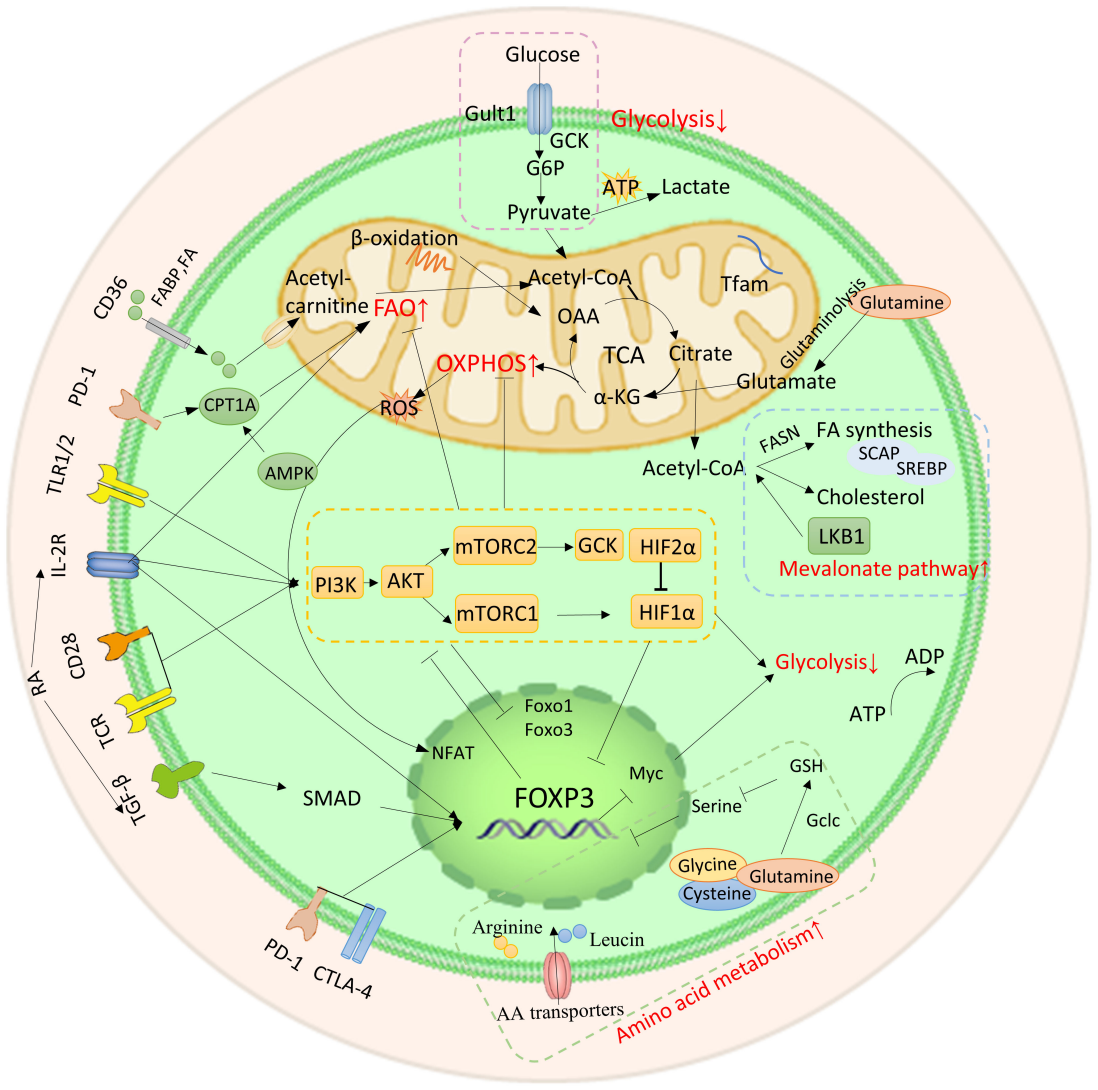
Metabolic and energy pathways reprogrammed by Tregs. After exTregs are reprogrammed into eTregs with immunosuppressive functions, Foxp3 regulates Treg cell metabolism by promoting FAO and inhibiting glycolysis. Meanwhile, amino acid metabolism, mevalonate metabolism and OXPHOS are promoted.

Metabolic and energy pathways play a crucial role in the survival and functioning of cells. Similar to other immune cells, Tregs in TME are activated and regulate their metabolic pathways to ensure biosynthesis and energy metabolism for their survival (**Figure 2**). The metabolic characteristics of the TME are often characterized by hypoxia, low glucose levels, and increased lactate production. Under conditions of inflammation, exTregs signal via the TLR1/2 pathway to enhance glycolysis by triggering the expression of Glut1 in a manner that relies on mTORC1. Simultaneously inhibiting Foxp3 expression and promoting glycolysis by affecting mTORC2 and cMyc. At the same time, the fatty acid oxidation pathway is inhibited. After exTregs are reprogrammed into eTregs with immunosuppressive functions, Foxp3 controls the metabolic activities of Treg cells by promoting FAO while restricting glycolysis by inhibiting the signaling pathways of c-Myc and mTORC2. Additionally, amino acid metabolism, mevalonate metabolism, and oxidative phosphorylation are improved (18–21).

#### 3.2.1 PI3K-Akt-mTORC1 axis

The PI3K-Akt-mTORC1 axis plays an important role in both signaling and metabolism. It is important to highlight that there exists a negative feedback regulatory mechanism between the PI3K-Akt-mTORC1 axis and Foxp3. This pathway can suppress the expression of Foxp3 through transcription factors. Conversely, increased expression of Foxp3 directly inhibits the pathway, resulting in reduced glycolysis and increased OXPHOS and FAO. The Tregs reprogramming process is regulated by the crucial PI3K-Akt-mTOR pathway, which includes important molecules such as AMPK, PTEN, CD28, and HIF-1α (21). Inhibiting PI3K and mTORC1, PTEN and AMPK serve as upstream regulators to enhance Foxp3 expression, thereby playing a significant role in the modulation of Tregs reprogramming (110, 111). The loss of the phosphatase PTEN results in a defect in reprogramming, causing immunosuppressive Tregs to transform into pro-inflammatory Tregs. CD28 promotes the activation of the PI3K-Akt-mTOR pathway (112). Additionally, HIF-2α inhibits HIF-1α and reduces the inhibition of Foxp3 expression by HIF-1α (113).

The mTOR is an important eukaryotic cell signal. Its expression has an impact on Treg formation, proliferation, and activity (114). It also plays an important role in autoimmune disorders and cancer. Sirolimus, often known as rapamycin, is a first-generation mTOR inhibitor. In autoimmune diseases, Treg depletion, mislocalization, or impaired function can be observed (115, 116). This can be improved by rapamycin (117). In contrast, the anticancer effect of rapamycin exceeds its immunosuppressive effect on tumor development (118). A plausible hypothesis is that rapamycin modifies the metabolic and protein synthesis of cancer cells to a greater extent than Tregs’ function (119–121). It’s also important to remember that rapamycin-mediated mTOR blockage may be particularly effective in certain forms of cancer that are preceded by autoimmune inflammation, such as mTOR-dependent cirrhosis that precedes hepatocarcinogenesis in patients with transaldolase deficiency (122, 123).

#### 3.2.2 Glycolysis

Under inflammatory conditions, TLR1/2 receptors on the surface of exTregs activate the PI3K-Akt-mTORC1 pathway, induce the expression of glucose transporter 1 (Glut1), and promote glycolysis (124). After the surface receptor CD28 is activated, it upregulates glucokinase (GCK) through the mTORC2 pathway and promotes glycolysis (125). IL-2R inhibits Foxp3 expression by inhibiting the transcription factors FOXO1 and FOXO3 through Akt (126–128). Nevertheless, the glycolysis inhibition in question arises when Foxp3 effectively dampens the PI3K-Akt-mTORC1 axis via feedback regulation (124). In parallel, the expression of the vital transcription factor Myc is suppressed by Foxp3, leading to the inhibition of glycolysis (129). After reprogramming of exTregs, Foxp3 expression is upregulated and the glycolysis pathway is inhibited.

#### 3.2.3 Amino acid metabolism

Treg reprogramming is largely dependent on amino acid metabolism. For instance, research conducted by Hao Shi et al. showed that arginine and leucine interact with the GTPases Rag A/B and Rheb 1/2. These proteins are necessary to maintain the adaptability of mitochondria and lysosomes, as well as for the expression of Treg suppressor gene signatures (130). Additionally, glutamine, glycine, and cysteine stimulate the expression of Foxp3 and enhance the immunosuppressive ability of Tregs through the activation of glutamate cysteine ligase (Gclc). At the same time, they inhibit serine, which negatively controls Treg function (131).

#### 3.2.4 Fatty acid oxidation and mevalonate metabolism

FAO is essential in promoting the reprogramming of Tregs and improving their immunosuppressive functions. The activation of IL-2R triggers the JAK-AMPK pathway, leading to the activation of FAO (132, 133). Simultaneously, PD-1 increases FAO by upregulating the expression of CPT1A and inducing the production of endogenous lipids. Moreover, the accumulation of free fatty acids (FFA) in TME activates fatty acid-binding protein (FABP) and CD36, thereby further promoting FAO. Notably, loss of CD36 weakens immunosuppressive function (21, 134).

The activation of the mevalonate pathway can be facilitated by liver kinase B1 (LKB1). LKB1 activates the mevalonate pathway by either upregulating the mevalonate gene or regulating intracellular cholesterol homeostasis (135). The mevalonate pathway metabolite geranylgeranyl pyrophosphate (GGPP) enhances STAT5 phosphorylation through IL-2 signaling, which is crucial for reprogramming Tregs to acquire immunosuppressive capabilities (135). Notably, the expression of mevalonate metabolic enzymes, as well as cholesterol synthesis and protein geranylgeranylation, is regulated by SREBP (sterol regulatory element binding protein)/SCAP (SREBP cleavage activating protein) signaling, thereby further supporting the mevalonate pathway (21, 136).

#### 3.2.5 Mitochondrial OXPHOS and mitochondrial complexes

The TME is rich in ROS, which is produced during mitochondrial OXPHOS. These ROS can activate NF-kB in Tregs and enhance the expression of the transcription factor NFAT. NFAT then binds with the non-coding sequence 2 (CNS2) of the upstream enhancer of the Foxp3 gene, leading to increased expression of Foxp3 and contributing to the reprogramming of Tregs (137). Furthermore, Foxp3 has the ability to enhance mitochondrial OXPHOS through the upregulation of genes and proteins involved in the electron transport system within mitochondria (138).

Mitochondrial electron transport chain (ETC) complexes, which are involved in OXPHOS, have been found to have an impact on Tregs function. The mitochondrial respiratory chain consists of five complexes. Alessia Angelin et al. conducted a study that revealed that mitochondrial complex I (also known as CI or NADH) could decrease the immunosuppressive function of Tregs (129). Similarly, Samuel E Weinberg et al. discovered that Tregs lacking complex III still maintained stable expression of Foxp3. However, there was a decrease in the expression of genes linked to Treg function (139). Additionally, in glucose-deprived TME, Tregs deficient in mitochondrial transcription factor A (Tfam) showed a decline in the expression of Foxp3, potentially attributed to increased methylation within the TSDR region of the Foxp3 site (140).

#### 3.2.6 Tumor microenvironment metabolites

Metabolites in the TME also affect Tregs function. As an active derivative of vitamin A, retinoic acid (RA) enhances the expression of Foxp3 by stimulating IL-2 to activate either the downstream JAK/STAT5 pathway or the downstream SMAD signaling pathway via TGF-βR (141, 142). However, there is some controversy surrounding this mechanism. In a different study, it has been observed that the absence of endogenous RA signaling actually enhances the suppressive ability and metabolic adaptation of Tregs. This enhancement was achieved through the stimulation of STAT5 and mTORC1 signaling (143).

### 3.3 Post-translational modification of Foxp3

**Figure 3:**
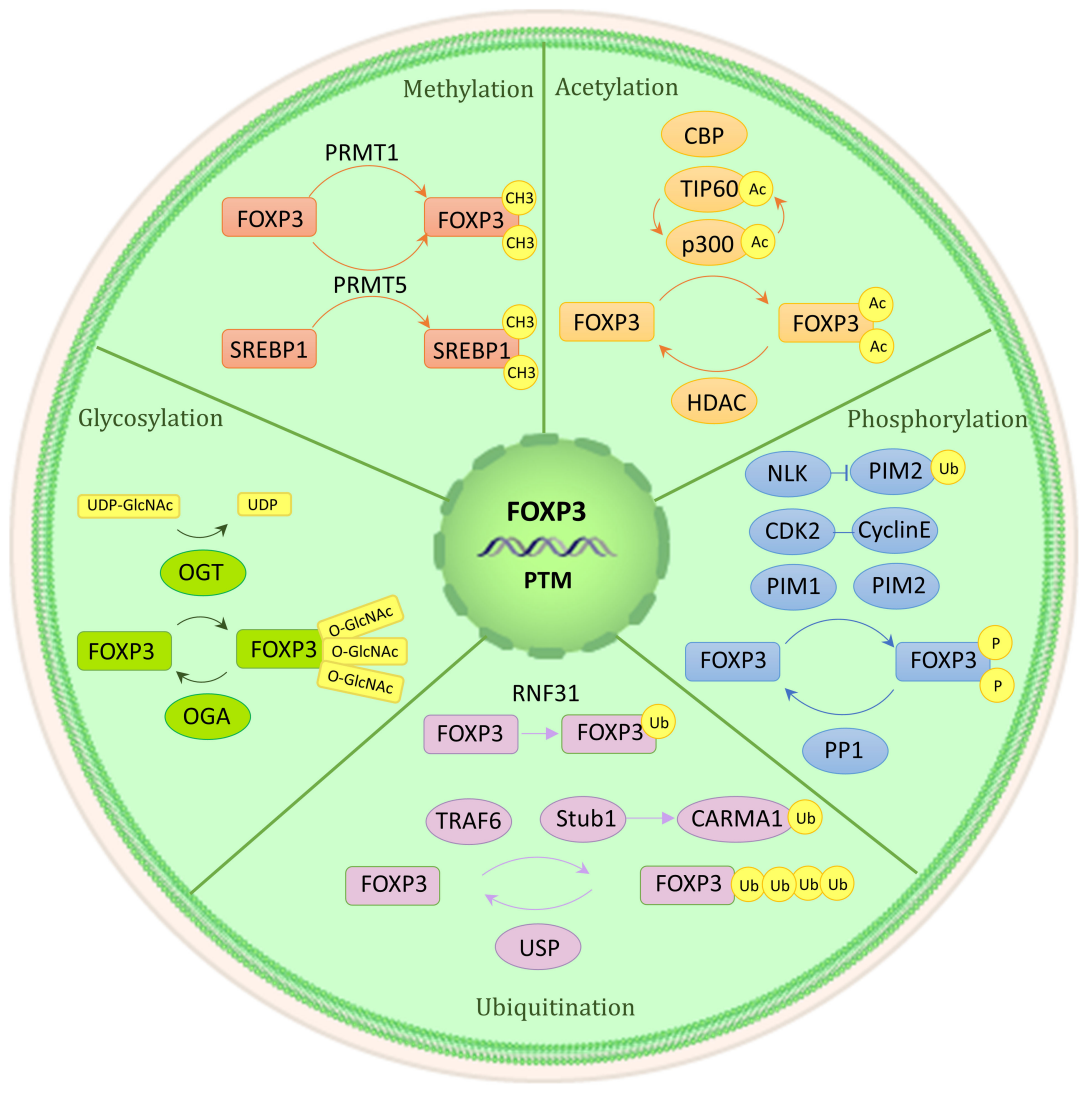
Post-translational modification of Foxp3. Numerous regulatory factors can post-translational modify Foxp3. A brief illustration is presented here, outlining their basic mechanism. Methylation (PRMT1 and PRMT5), acetylation and deacetylation (CBP, p300, TIP60, HDAC), phosphorylation (CDK2, PIM1, PIM2, NLK, PP1), ubiquitination, deubiquitination, and SUMO (RNF31, STUB1, TRAF6, USP1), glycosylation (OGT, OGA).

Proteins’ posttranslational modifications (PTMs) are important for linking cellular signaling to functional characteristics. PTM refers to the enzymatic process that alters proteins after their synthesis. The Foxp3 protein consists of three functionally significant domains: the N-terminal domain, the zinc finger and leucine zipper regions, and the C-terminal forkhead domain. The stability of Foxp3 expression and Tregs’ immunosuppressive role are facilitated by methylation, acetylation, and glycosylation of the protein (**Figure 3**). On the other hand, the effects of phosphorylation and ubiquitination are more complex, having both positive and negative impacts (9, 22, 23).

#### 3.3.1 Methylation

Protein methylation is the enzymatic transfer of a methyl group to certain residues of a protein, such as lysine or arginine. Additional residues that can undergo methylation include histidine, cysteine, and asparagine (144). The methylation of the Foxp3 is significantly impacted by the arginine methyltransferase (PRMT) family members, including PRMT1 and PRMT5 (22). Specifically, PRMT1 adds asymmetric dimethylarginine to Arg-48 and Arg-51 of Foxp3. The immunosuppressive ability of Tregs is greatly reduced when the methylation of these two sites is inhibited (145). Similarly, symmetric dimethylarginines on Foxp3’s Arg-27, Arg-51, and Arg-146 are catalyzed by PRMT5. Among these modifications, methylation silencing on Arg-51 can restrict the inhibitory function (146), potentially because RPMT5 regulates the production of the IL-2R’s γ chain (CD132) (147). Furthermore, PRMT5 can also methylate SREBP1, which can regulate cholesterol biosynthesis, as mentioned previously (148).

#### 3.3.2 Acetylation and deacetylation

Acetylation is a PTM primarily observed on histones and other cellular proteins. Acetyl groups are added to proteins through histone acetyltransferase (HAT), now referred to as acetyltransferase (KAT), which enhances Foxp3 expression and immunosuppression. Conversely, histone deacetylase (HDAC), now known as lysine deacetylase (KDAC), catalyzes the opposite reaction, negatively regulating Foxp3 expression and impairing the immunosuppressive function of Tregs (22, 149). The coordination of Treg formation, function, and reprogramming is greatly aided by these enzymes.

The main HATs involved in Treg acetylation are p300, CBP, and TIP60. Acetylation of Foxp3 by p300 increases the levels of Foxp3 and prevents its degradation by proteases (150). Three acetylation sites, including K31, K262, and K267, have been found using mass spectrometry investigation (151). The loss of p300 leads to a decrease in the immunosuppressive ability of Tregs and an enhancement of anti-tumor immunity (152, 153). CBP, a paralog of p300, also has a significant function in controlling the activity of Treg in specific inflammatory conditions (154). TIP60 and p300 collaborate to control the functioning of Foxp3. p300 forms an interaction with TIP60 and facilitates the autoacetylation of TIP60 at K327. Such adjustment elevates the robustness of the TIP60 protein and accelerates the acetylation process of Foxp3. Similarly, TIP60 also amplifies p300 acetylation and the activity of HAT. When TIP60 and p300 are both present (e.g., at K179 and K227 sites), acetylation of Foxp3 becomes more pronounced (155). Furthermore, if the forkhead domain of Foxp3 undergoes mutation, it disrupts the binding between Foxp3 and TIP60, leading to a reduction of Tregs’ function. Still, this can be reversed by allosteric modifiers that strengthen the interaction between Foxp3 and TIP60 (156, 157).

There are four distinct categories into which HDACs can be classified. HDACs of class I, class II, and class IV function through the dependence on zinc ions (Zn2+). Conversely, sirtuins, known as class III HDACs, make use of nicotinamide adenine dinucleotide (NAD) as a coenzyme (158). For the execution of their role, Class I HDAC1 and HDAC2 attach themselves to the N-terminal section of Foxp3 (159). HDAC3 knockout mice have been found to have significantly impaired immunosuppressive function (160). Class II HDACs include HDAC6,7,9,10. The lack of HDAC6 enhances the expression of numerous genes associated with Tregs, such as IL-10, Lag3, and STAT3, thereby greatly enhancing the suppressive capability of Tregs (161, 162). In the TCR signaling scenario, the separation of HDAC9 from Foxp3 disrupts STAT5 acetylation, which subsequently hinders the functioning of Foxp3 (163–165). On the other hand, HDAC10 facilitates the deacetylation of Foxp3 at Lys-31 (166). The specific mechanism behind these processes is still unknown, though. Class III SIRT1 is a NAD-dependent enzyme that negatively regulates TIP60 acetylation (167, 168). This regulatory process can be disrupted by mammalian sterile 20-like kinase 1 (MST1) (150, 169). Additionally, Class IV Zn2+-dependent HDAC11 can co-associate with Foxp3 and contribute to its deacetylation (165, 170).

#### 3.3.3 Phosphorylation

Protein kinases engage in phosphorylation to covalently attach a phosphate group to a protein’s serine (S), threonine (T), or tyrosine (Y) residue. This process is reversible. Among them, cyclin-dependent kinase (CDK2), proto-oncogene serine/threonine protein kinase (PIM1, PIM2) and protein phosphatase 1 (PP1) negatively regulate Foxp3 expression. Kinase NLK positively regulates Foxp3 expression (22).

CDK2, PIM1, and PIM2 each inhibit Treg immunosuppressive function through distinct mechanisms. Four cyclin-dependent kinase motifs (Ser/Thr-Pro) are present in the N-terminal repressor domain of Foxp3. Cyclin E interacts with CDK2 and phosphorylates Foxp3 at these sites, resulting in decreased stability of Foxp3 protein and reduced suppressive function of Tregs (171, 172). In contrast, PIM1 and PIM2 have no effect on the decrease in stability of Foxp3. Instead, they impact its activity of binding to DNA and interacting with other co-factors. The crucial role of DNA binding for Foxp3 is fulfilled by its forkhead domain (FHD). PIM1 phosphorylates Foxp3 at Ser-422, which is situated in the C-terminal region of FHD (173). Foxp3’s interaction with co-factors such as HDAC7, TIP60, and Eos depends on its N-terminal region. PIM2 phosphorylates Ser-33 and Ser-41 within the N-terminal region of Foxp3 (174, 175). Additionally, lymphocyte-specific protein tyrosine kinase (LCK) phosphorylates Foxp3 at Tyr-342, which inhibits cell invasion. However, the functional impact of LCK phosphorylation on Foxp3 remains unclear (176).

How PP1 affects Foxp3 expression and Treg function remains a topic of debate (9, 23, 177). According to Hong Nie et al.’s study, during inflammatory circumstances, PP1 dephosphorylated Foxp3 at Ser-418 of FHD, leading to a reduction in Foxp3 expression (178). On the other hand, Nemo-like kinase (NLK) was found to promote Foxp3 expression. NLK phosphorylates Foxp3 and prevents its degradation by the ubiquitin ligase Stub1 (179, 180).

#### 3.3.4 Ubiquitination, deubiquitination, and small ubiquitin-like modifier

Ubiquitination, a process mediated by a sequence of enzymes, denotes the covalent attachment of ubiquitin to target proteins. This intricate procedure necessitates the synergies of three distinct ubiquitin enzymes: E1 ubiquitin-activating enzyme, E2 ubiquitin-conjugating enzyme, and E3 ubiquitin ligase. Substrate proteins can undergo monoubiquitination or polyubiquitination. Monoubiquitination takes place when a solitary ubiquitin molecule binds to a lone Lys residue of the target protein, whereas polyubiquitination entails the concurrent labeling of various Lys residues of the target protein by a solitary ubiquitin molecule (181–183). The three enzymes responsible for ubiquitinating Foxp3 are RNF31, Stub1, and TRAF6, each serving different functions.

RNF31, a crucial E3 ligase found in the LUBAC complex (linear assembly complex of ubiquitin chains), holds a vital function in facilitating the monoubiquitination of Foxp3. This particular protein actively participates in numerous immune signaling pathways and carries substantial implications in Tregs (184–188). In addition to regulating TCR signaling, RNF31 also facilitates Foxp3 monoubiquitination, resulting in heightened Foxp3 expression and improved immunosuppressive function in Tregs (189).

Stub1 and TRAF6 are involved in the polyubiquitination of Foxp3. Stub1 mediates K48 polyubiquitination at multiple sites, leading to the degradation of Foxp3 and a reduction in Treg suppressive activity (180). Additionally, Stub1 can also mediate K27 polyubiquitination on CARMA1 in the CBM complex, so aiding in the NF-κB’s activation (190). On the other hand, TRAF6, a cyclic E3 ligase, is responsible for mediating inflammation-related signaling (191). K63 polyubiquitination on Foxp3 at Lys-262 is mediated by TRAF6, but the exact mechanism of its impact on Foxp3 still requires further investigation (192).

Deubiquitinase (DUB) can reverse the reversible process of ubiquitination. DUB removes ubiquitin from substrate proteins (193). Several proteins, including USP7, USP21, USP22, and USP44, have been discovered to directly interact with Foxp3. USP7 prevents the ubiquitin-dependent degradation of Foxp3 protein by removing the K48 polyubiquitination tag. Additionally, it promotes the interaction of TIP60 with Foxp3, thereby preserving the level of expression of Foxp3 and the immunosuppressive ability of Treg (194). Similarly, USP21, USP22, and USP44 also deubiquitinate Foxp3, preventing its degradation (195–199).

Small ubiquitin-like modifiers are small molecules that can be covalently bound to other proteins through a cascade reaction mediated by enzymes. This process is known as SUMO. SUMO does not involve the proteolysis of targeted proteins but rather plays a crucial part in regulating diverse biological processes. It is important to note that the SUMO process is reversible. For instance, ROS induced by TCR signaling controls the protein stability of the deubiquitinating enzyme SENP3, thereby mediating the deubiquitinating modification of the Foxp3 transcription factor BACH2 and its activity in maintaining the immunosuppressive function of Tregs (200).

#### 3.3.5 Glycosylation

Glycosylation is a type of PTM that involves attaching glycans to proteins. Observations demonstrate a direct link between the suppressive activity of Tregs and glycosylation, indicating a positive correlation (201, 202). O-GlcNAc glycosylation modification specifically refers to the addition of a single monosaccharide modification to the serine and/or threonine residues of a protein. O-GlcNAc transferase (OGT) and O-GlcNAcase (OGA) are enzymes that catalyze this modification in opposite directions. There are several O-GlcNAcylation locations in Foxp3, which facilitate the expression of Foxp3 by regulating the IL-2/STAT5 pathway following glycosylation (203). It is significant to highlight that the glycosylation of c-Rel, another transcription factor, has been demonstrated to diminish its interaction with Foxp3 and inhibit Foxp3 expression (204). Moreover, O-GlcNAcylation could oppose ubiquitination, resulting in the enhancement of Foxp3 protein stability (205).

## 4 Immunotherapy targeting Treg reprogramming

With the advancement of cancer immunotherapy and the in-depth exploration of the TME, cancer treatment has experienced a revolution. In TME, Treg is a major therapeutic target. There are currently seven immunotherapies targeting Tregs, such as (1) depleting TI-Tregs, (2) preventing the migration of Tregs into tumors, (3) sensitizing TI-Tregs to inhibitory receptor blockade, (4) targeting costimulatory signals on TI-Tregs, (5) targeting Treg cytokine secretion, (6) altering Treg stability, (7) disrupting exTreg reprogramming (206). As shown in **Table 1**, we summarized potential targets that may act on Tregs reprogramming.

**Table 1:** Targeting potential targets of Tregs reprogramming.

Method	Target	Drug	Mechanism	Combination therapy	References
Signal molecules and signaling pathways	MALT1/CBM complex	Mepazine	The disruption of Malt1 caspase activity by mepazine leads to impaired CTLA-4 expression and suppressive function in Tregs associated with improved tumor control.	Combination therapy of MALT1 inhibitor- meprazine and anti-PD-1 ICI.	(207, 208)
	IL-2R	Daclizumab	The binding of IL-2 and IL-2R leads to the phosphorylation and activation of transcription factor STAT5, promoting Foxp3 expression and Treg immune suppression.	IL-2 has been used in combination with adoptive cell therapy (ACT) for the treatment of melanoma.	(69, 71)
	S1P	PF-429242, FTY720	The sphingosine-1-phosphate receptor (S1PR1) can activate the PI3K-AKT-mTOR1 pathway, disrupting immune suppression.	N/A	(209–211)
	PTEN	VO-OHpic	PTEN can block the PI3K-Akt-mTOR1 pathway and disrupt Treg immunosuppression.	N/A	(111, 212)
	GITR	DTA-1	GITR becomes an attractive target for cancer immunotherapy after the agonistic anti-GITR antibody DTA-1 is shown to block the suppressive effects of Tregs.	Synergistic antitumor responses by combined GITR activation and sunitinib in metastatic renal cell carcinoma.	(91, 213)
	TGF-β	Ibalizumab	Blocking TGF-β signaling in Tregs remodels the tumor microenvironment and inhibits cancer progression.	Manganese synergizing anti-TGF-β/PD-L1 bispecific antibody YM101 to overcome immunotherapy resistance in non-inflamed cancers.	(50, 214, 215)
	OX40	Rocatinlimab, amlitelimab	OX40 is also able to activate the Akt and STAT5 pathways in Tregs, resulting in decreased Foxp3 expression levels.	Combined OX40 agonist and PD-1 inhibitor immunotherapy improves the efficacy of vascular targeted photodynamic therapy in a urothelial tumor model.	(95, 216, 217)
	AHR	CH223191	AHR targeting in IDO/TDO-expressing tumors counteracts a regulatory T cell/macrophage suppressive axis and synergizes with immune checkpoint blockade to hinder tumor growth.	N/A	(101, 218)
	IDO	1-MT	IDO signaling inhibits Akt, upregulates transcription factor Foxo3a, and promotes Treg reprogramming into an immunosuppressive phenotype.	The application of IDO inhibitors combined with RT may have a synergistic effect by relieving immunosuppression.	(106, 219)
	CCR8	ML604086	The CCR8-CCL1 pathway enhances the immunosuppressive ability of Treg by upregulating the expression of FOXP3, CD39, and IL-10.	Therapeutic depletion of CCR8+ tumor-infiltrating regulatory T cells elicits antitumor immunity and synergizes with anti-PD-1 therapy.	(108, 109, 220, 221)
Metabolic reprogramming	Glycolysis	Metformin	Metformin treatment leads to mTORC1 activation and metabolic reprogramming of Tregs toward glycolysis, which leads to reduced inhibitory function and induces apoptosis of Tregs.	Metformin promotes antitumor immunity via endoplasmic-reticulum-associated degradation of PD-L1.	(222, 223)
	OXPHOS	Rotenone, oligomycin	Rotenone and oligomycin can inhibit the oxidative phosphorylation of Tregs in tumors, thereby disrupting their immunosuppressive function. Their anti-cancer properties have been proven.	N/A	(224)
	FAO	Soraphen A, pioglitazone	Soraphen A and pioglitazone can inhibit the FAO of Tregs in tumors, thereby disrupting their immunosuppressive function. Their anti-cancer properties have been proven.	N/A	(225, 226)
	Mevalonate pathway	Simvastatin	Simvastatin can inhibit the mevalonate pathway of Tregs in tumors, thereby disrupting their immunosuppressive function. Their anti-cancer properties have been proven.	N/A	(227, 228)
	HIF-1α	CDMP-TQZ, YC-1, PX-478, echinomycin	The absence of HIF-1α in Tregs increases mitochondrial metabolism and immune suppression, but reduces migration ability, ultimately leading to a decrease in the number of Tregs in the tumor.	N/A	(229–233)
	Tfam	N/A	Tfam deletion in Tregs reduces intratumoral Tregs, their Foxp3 expression, and suppressive function, leading to better tumor control.	N/A	(140)
	Mitochondrial respiratory chain complex III	N/A	Genetic inactivation of mitochondrial respiratory chain complex III can lead to loss of Treg suppressive function and marker genes without altering Foxp3 expression. Ultimately, it can inhibit tumor growth.	N/A	(139)
	CD36	AP5055	CD36 deletion in Tregs decreases intratumoral Tregs, and their suppressive function, while promoting their production of IFN-γ and TNF in association with improved tumor control.	Combination of anti-CD36 and anti-PD-1 treatment or anti-PD-1 treatment in Treg-specific CD36-deficient mice resulted in improved tumor control and prolonged survival.	(134, 234)
	PRMT1	Doxycycline	PRMT1 promotes arginine methylation of Foxp3, thereby promoting the inhibitory function of Tregs.	The combination therapy of PRMT1 inhibitors and anti-PD-1 antibodies enhances the therapeutic effect of anti-tumor therapy *in vivo.*	(145, 235)
	PRMT5	DS-437	The pharmacological inhibition of PRMT5 by DS-437 inhibits the methylation of FOXP3 and reduces the immunosuppressive function of Tregs.	N/A	(146)
	p300	C646	Conditional Treg deletion or pharmacological inhibition (C646) of p300 increases the intratumor Teff: Treg ratios and led to tumor growth control.	The p300/CBP inhibition enhances the efficacy of programmed death-ligand 1 blockade treatment in prostate cancer.	(152, 236)
Posttranslational modification	TIP60	Small molecule inhibitors of Usp7(Usp7i), including P5091 and P0217564	The deubiquitinase Usp7 controls the level of histone acetyltransferase Tip60 and to a lesser extent controls the level of Foxp3. Gene deletion or pharmacological inhibition of Usp7 can impair the immunosuppressive function of Tregs.	The combination therapy of anti-PD-1 monoclonal antibody and Usp7i has better anti-tumor effects than Usp7i alone.	(195)
	PP1	PPP1R11	The loss of PPP1R11 induced resistance toward Treg-mediated suppression in T cells as measured by gene and protein expression of T cell stimulation-induced cytokines IL-2 and IFN-γ.	N/A	(237)
	RNF31	HOIPIN-8	RNF31 can lead to increased Foxp3 expression and enhanced immunosuppressive function of Treg cells.	N/A	(189, 238)
	TRAF6	Traf6i(6877002)	TRAF6 inhibitor boosts antitumor immunity by impeding regulatory T cell migration in the Hepa1-6 tumor model.	OX40-TRAF6 axis promotes CTLA-4 degradation and is a potential therapeutic target for the improvement of T-cell-based immunotherapies.	(239–241)

NA: Not Applicable.

## 5 Conclusion

After extensive research, there has been a better understanding of Tregs. In the inflammatory microenvironment, Tregs are influenced by signaling molecules, metabolic conditions, and Foxp3 post-translational modifications. As a result, they undergo reprogramming and transform into immunosuppressive Tregs, which enhances immunosuppressive ability. Tregs are now a prominent target in the realm of cancer immunotherapy. By influencing the reprogramming of Tregs, it is possible to effectively reduce their immunosuppressive function, mitigate the negative effects of Treg reprogramming on immunotherapy in inflammatory conditions, and partially prevent the severe autoimmune consequences that may arise from Treg depletion therapy.

Existing technology and approaches are making great efforts to address the inadequacies of various immunotherapies. Considering the current negative consequences of Treg depletion-induced autoimmunity, there are a number of ways to effectively induce tumor immunity by targeting specific Tregs without triggering severe autoimmune reactions. The first one is to selectively target effector Treg cells in tumor tissues, thereby retaining naïve Tregs in other tissues that are needed to prevent autoimmunity. The second aims to adjust the extent and duration of Treg depletion. The third method is to inject Treg-depleting antibodies directly into the tumor tissue. In addition, when Tregs depletion is used in combination with other immunotherapies, Tregs should be depleted or their suppressive activity should be weakened before other treatments (such as immunosuppressants and vaccination) (242).

In targeted Tregs reprogramming, multi-omics approaches combined with novel computational tools can be applied to target various signals and molecules on Tregs (243–245). It enables a better understanding of the gene regulatory networks regulating Treg function, as well as the identification of additional biomarkers that define Tregs function in the TME. This could go a long way toward new therapies targeting Tregs without affecting immune homeostasis in various cancers (206).

The drawback of targeted post-translational modifications is that they are difficult to selectively target because they are present in both cancerous and normal cells on a large scale. The issue with metabolic reprogramming that targets Tregs is the same (21). Furthermore, targeting post-translational modifications is more common in hematological malignancies and lacks application in solid tumors. In recent studies, proteolytic targeting chimeras (PROTACs) have been used as an innovative technology to target parts of proteins that could not be targeted before. They affect Tregs function by connecting target protein ligands and E3 ubiquitin ligase ligands, promoting rapid ubiquitination and consequent degradation of target proteins (246–248).

However, there are still some issues that we need to explore further. (1) Are there other mechanisms affecting Tregs reprogramming? (2) Can tumor-specific Treg-targeted therapy be achieved without affecting tissue Tregs? (3) How much impact do different inflammation and cancer types have on Tregs? We believe that in the future, with continued in-depth research on Tregs, significant progress will be made in the field of tumor immunotherapy, benefiting millions of cancer patients.

## Author contributions

JL: Conceptualization, Writing – original draft, Writing – review & editing. BZ: Conceptualization, Writing – original draft, Writing – review & editing. GZ: Conceptualization, Writing – original draft. DS: Conceptualization, Writing – original draft, Writing – review & editing.
